# Psychological Distress Among Cancer Patients During COVID-19 Pandemic in the World: A Systematic Review

**DOI:** 10.3389/fpsyg.2021.682154

**Published:** 2021-09-28

**Authors:** Zohre Momenimovahed, Hamid Salehiniya, Fatemeh Hadavandsiri, Leila Allahqoli, Veronika Günther, Ibrahim Alkatout

**Affiliations:** ^1^Department of Reproductive Health, Qom University of Medical Sciences, Qom, Iran; ^2^Social Determinants of Health Research Center, Birjand University of Medical Sciences, Birjand, Iran; ^3^Cancer Research Center, Shahid Beheshti University of Medical Sciences, Tehran, Iran; ^4^School of Public Health, Iran University of Medical Sciences (IUMS), Tehran, Iran; ^5^Kiel School of Gynecological Endoscopy, University Hospitals Schleswig-Holstein, Kiel, Germany

**Keywords:** COVID-19, cancer, anxiety, depression, distress, fear

## Abstract

**Aim:** Patients with malignancies, experience high rates of psychological distress. Fear of Corona-infection combined with the interruptions in some treatment programs might affect the psychological health of cancer patients. This review study was conducted to investigate the psychological distress among cancer patients during COVID-19 pandemic to offer system-adapted individual solutions.

**Materials and methods:** To identify the psychological distress of cancer patients, a comprehensive search was carried out in PubMed, Web of Science, and Scopus. English language and original articles were included in this study. Articles that addressed any psychological distress among cancer patients during COVID-19 pandemic were included.

**Results:** At first 1,410 articles, were included in the study. After removing duplicate articles and reviewing the title and abstract, 55 articles were selected for the review. The findings of this study revealed COVID-19 greatly affects psychological health of cancer patients. Fear of COVID-19, fear of disease progression, disruption of oncology services, cancer stage, and immunocompromised status were the most common causes of psychological distress in oncology patients which can influence patients' decisions about treatment.

**Conclusion:** The COVID-19 related anxiety is an expected reaction to the current situation. Although psychological distress affects many people, it can confuse cancer patients to the point that they refuse to continue treatment for the fear of infection and worsening of their condition. Since the end of this pandemic is unknown, this action can endanger the health and prognosis of this group of patients, so it seems that using psychological interventions and intensive counseling in the current situation is one of the main priorities for cancer patients.

## Introduction

From March 2020, in a short time, the lives of people all around the world underwent extensive changes following the occurrence of a new disease called COVID-19 (Spinelli and Pellino, 2020). In response to this pandemic, treatment centers made significant changes in the management and treatment of patients to prevent overcrowding caused by emergency and non-emergency patients (Kaufman et al., 2020). Although the impact of COVID-19 pandemics in different geographical areas is not clear, delays in diagnosis and treatment due to concerns about coronavirus, restrictions on medical facilities such as reduced non-emergency hospitalization and reduced access to physicians can lead to increase the stage of the disease, weaker prognosis and higher mortality, and ultimately has adverse effects on patients' psychological health (Maringe et al., 2020; Sharpless, 2020). Studies have shown that patients with malignancies, experience higher rates of distress, anxiety, and depression than the general population, and the slower the course of treatment, the higher the distress would be (Pitman et al., 2018; Tsaras et al., 2018; Slimano et al., 2020). Efforts have long been made to reduce the physical and psychological distress of various patients, especially sensitive and vulnerable oncology patients. However, facing an unprecedented situation this requires accurate knowledge of the effects of pandemics on the occurrence of psychological disorders among cancer patients. So this review study was designed and conducted to investigate the psychological distress, type of psychological problem, prevalence, causes and effects of these problem on cancer patients during COVID-19 pandemic.

## Materials and Methods

### Search Strategy

The present study was conducted to investigate the effects of COVID-19 on the fear and anxiety of cancer patients. A comprehensive search was conducted in databases including to find related articles published until the end of June 2021. The keywords such as mental disorder, distress, anxiety, depression, psychiatric disorder, psychiatric illness, cancer, tumor, oncology, malignancy, and neoplasms, Covid-19, coronavirus, SARS-CoV-2 and 2019-nCoV were used to perform the search. In order to make the search more comprehensive, a manual and bibliographic search was also performed (references of included article). All retrieved articles were entered in Endnote-X7 software. The search strategy of this study is presented in Appendix 1.

### Inclusion Criteria

Inclusion criteria were, being original article, part or all of the study samples should be cancer patients, English language, and one of the objectives of the study should be measurement of the psychological distress among cancer patients during COVID-19 pandemic.

### Exclusion Criteria

Non-peer reviewed articles, Commentaries, editorials, systematic review, opinion statements, practice guidelines, case series or case reports were excluded from the study.

### Data Extraction

Articles were searched by one of the researchers (HS) and they were entered into Endnote software. The inclusion and exclusion of articles based on the title and abstract were done independently by two researchers (ZM, HS). At this stage, articles that did not meet the inclusion criteria were removed, and the full text of all articles that met the inclusion criteria were prepared and reviewed. By examining the articles in detail, their results were extracted. This review was done based on the PRISMA guidelines. Data including author and year of the study, location, design, sample size, cancer type, participant age, type of questionnaire and main results were extracted by two independent investigators, as appropriate.

## Results

### Characteristics of the Selected Studies

**Figure 1:**
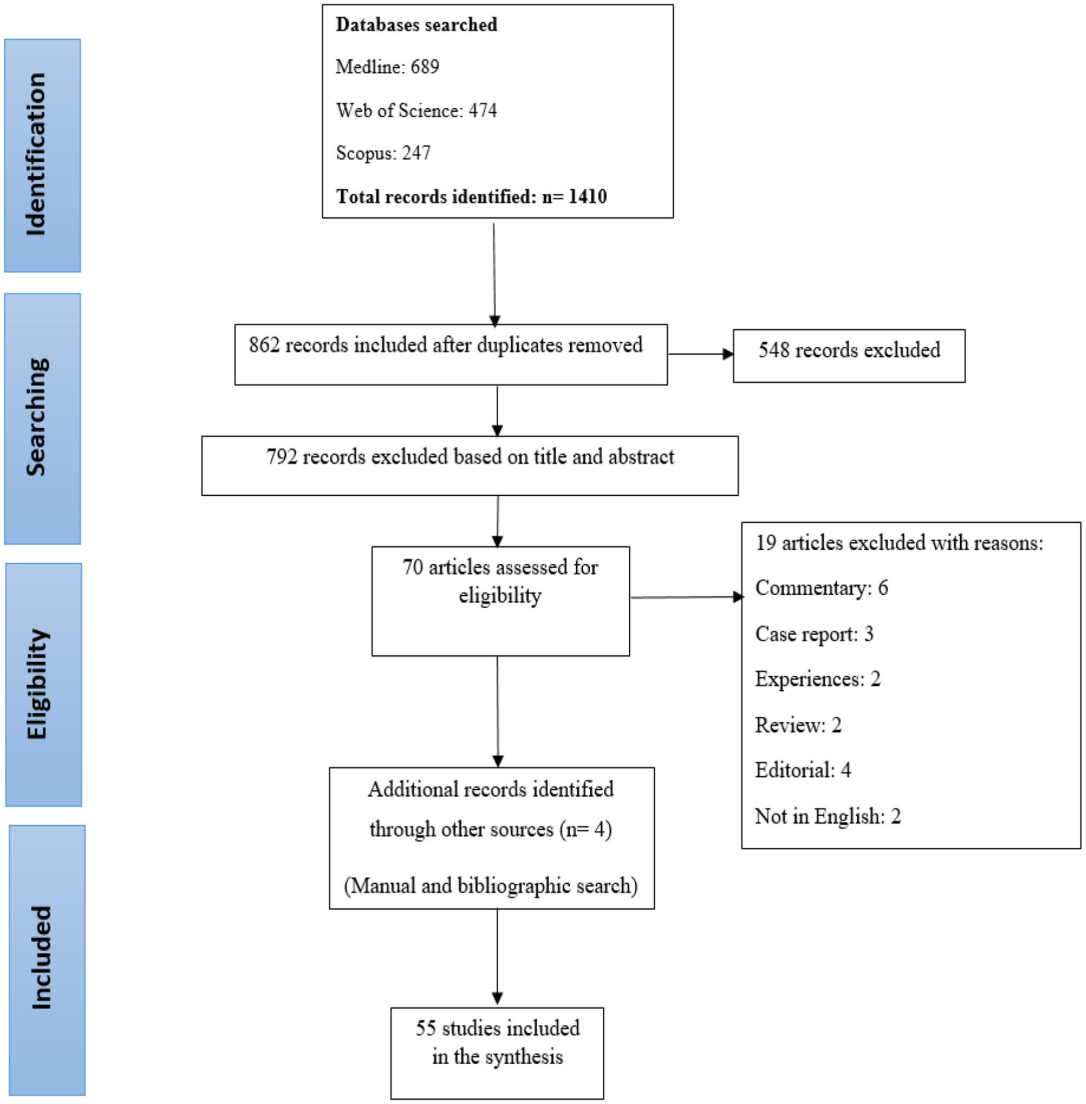
Study flow diagram.

At first, 1,410 articles, were included in the study. After removing duplicate articles using Endnote software, 862 articles were selected for the review. After reviewing the title and abstract, 792 articles were not related to the purpose of the study and did not meet the study criteria and therefore, were removed. Also, 19 articles were deleted for other reasons (Commentary: 6, Case report: 3, Experiences: 2, Review: 2, Editorial: 4, Not in English: 2); the references of all included articles were reviewed manually and finally 55 articles selected for the review (Figure 1).

### Prevalence of Psychological Disorders Among Cancer Patients

COVID-19 greatly affects not only the physical health, but also the psychological health of people (Ornell et al., 2020; Sweeney and Ahlstrom, 2020; Sun and Li, 2021; Wong et al., 2021). Fear and anxiety are the psychological response of many people to COVID-19 pandemic, including cancer patients, and these people experience a significant psychological burden at the current time. Depression, isolation, anxiety, insomnia and fear are the most important psychological problems of cancer patients during the COVID-19 pandemic that have been mentioned by researchers in various studies (Chen et al., 2020, 2021a; Gallagher et al., 2020; Swainston et al., 2020; Wang et al., 2020a; Büntzel et al., 2021; Levy et al., 2021; Rodrigues-Oliveira et al., 2021; Wong et al., 2021).

In a study, Cui et al., examined the extent of psychological distress in cancer patients. They stated that a significant proportion of cancer patients were suffering from depression (51.2%), anxiety (62.8%), insomnia (51.2%), and PTSD (35.5%), and the level of psychological distress in this group was significant compared to the frontline nurses fighting the pandemic (Cui et al., 2020). Miaskowski examined 187 cancer patients for psychological problems and identified depression (71.2%), anxiety (78.0%), sleep disorders (78.0%), fatigue (55.9%), and cognitive weakness (91.5%) in them (Miaskowski et al., 2020). A study in France examined the psychological distress among four vulnerable groups, including breast cancer patients. The researchers concluded that peri-traumatic distress in these patients is at a high level, which exposes them to post-traumatic stress disorder (Chaix et al., 2020). According to a study from Spain, 33.5% of patients experienced clinical stress levels (Yélamos Agua et al., 2021). Chaix in a cross-sectional study reported Prevalence of psychological distress was 34% for breast cancer patients (Chaix et al., 2020).

The results of a study from Wuhan, China showed anxiety in 67.5% of cancer patients, and these patients experienced higher levels of depression than anxiety (74.5%) (Chen et al., 2020). A study in China that examined 6,213 cancer patients showed that 23.4% of the samples were suffering from depression, 17.7% from anxiety and 9.3% from post-traumatic stress disorder (Wang et al., 2020a). A cross sectional study from Iran reported 61.4 % of cancer patients had moderate to severe anxiety (Dehghan et al., 2021). A study of 77 patients with lymphoma using the Hospital Anxiety and Depression Scale (HADS) showed that 36% of the samples had anxiety, 31% had depression, and 36% had post-traumatic stress disorder (Romito et al., 2020). In their study, Frey et al., found concern in 88.6% of patients with ovarian cancer during COVID-19 pandemic, so they reported that during this period, anxiety and depression affected a significant number of patients (Frey et al., 2020). The results of a study from Singapore identified fear of widespread spread of the virus in 66% of patients and anxiety in 19.1% of them (Ng et al., 2020).

### Related Factors

The occurrence of psychiatric disorders led researchers to investigate its associated factors. In this regard, the results of a study showed that most cancer patients experienced at least one stressor during the COVID-19 pandemic. These stressors are associated with higher rates of anxiety, depression, and insomnia (Massicotte et al., 2021). The findings of a cross-sectional study conducted by Swainston et al. showed that the concerns that patients experienced after the onset of COVID-19 pandemic had a psychological effect on them. The researchers found that disruption of oncology services is associated with the COVID-19 pandemic and higher levels of anxiety and depression (Swainston et al., 2020). In a study, Bargon et al. concluded that, among 1,051 patients with breast cancer, there was a significant decline in psychological well-being, which could be due to having less contact with physicians (Bargon et al., 2020). Other studies have confirmed the results of previous studies by stating that high levels of depression, changes in treatment plans, and concerns about not seeing the physicians are the most important reasons for oncology patients' high level of anxiety during the COVID-19 pandemic (Chen et al., 2020, 2021a; Swainston et al., 2020; Gultekin et al., 2021; Hamlish and Papautsky, 2021; Nardone et al., 2021; Yildirim et al., 2021). In a study, Chapman et al., examined the effect of COVID-19 on job security and emotional functioning of breast cancer patients, and concluded that the existence of threat against job security was associated with depression in individuals. According to this study, employment and more intense mental engagement were associated with more anxiety and depression and poorer cognitive function (Chapman et al., 2020). According to studies, the immunocompromised people who are in a state of active treatment, quarantine and loneliness, or experience delay in any part of their treatment process have a higher level of anxiety (Chen et al., 2020, 2021a; Frey et al., 2020; Chia et al., 2021; Papautsky and Hamlish, 2021). A study from US reported delay in cancer care was associated with a 4-fold increased rate of anxiety (Chen et al., 2021b). In addition, the presence of pre-existing psychological problems and alcohol consumption was associated with the occurrence or exacerbation of psychological distress during the pandemic (Wang et al., 2020a; Gultekin et al., 2021; Koinig, 2021). According to Frey's study, being under the age of 65, planning for cancer treatment or surgery, being immunosuppressed, and using telemedicine exacerbate anxiety and are associated with depression (Frey et al., 2020). Hamlish and Papautsky reported white participants experience more stress than blacks, and often worry about interruptions or delays in care, treatment, worsening of the disease, COVID-19, and general health (Hamlish and Papautsky, 2021). Studies show that patients' gender and age can also lead to the development of psychological disorders, as women and young patients are more prone to anxiety and stress disorder after trauma (Frey et al., 2020; Romito et al., 2020; Koinig, 2021; Pigozzi et al., 2021; Yélamos Agua et al., 2021). On the other hand, in addition to the type of disease, its stage also determines the level of psychological disorders. For example, patients with advanced refractory cancer experience higher levels of distress (Zhang et al., 2021). Late stage of cancer is associated with higher anxiety compared to the lower stage (Chen et al., 2020). Gultekin in a cross-sectional study found gynecological cancer patients experienced significant worry about progression of their disease due to modifications of care, delay or cancellation of oncology treatment or follow-up in the COVID-19 pandemic (Gultekin et al., 2021).

In addition to anxiety and depression, fear also affects a significant number of cancer patients during the COVID-19 pandemic (Schellekens and van der Lee, 2020; Chia et al., 2021). Fear of disease progression, fear of disease recurrence and comorbidity along with depression and insomnia causes new challenges for patients (Chen et al., 2020, 2021a; Mahl et al., 2020; Musche et al., 2020; Sigorski et al., 2020; Massicotte et al., 2021; Yang et al., 2021). Fear, which can be examined at different levels and from different aspects, can threaten a person's health in this crisis. Fear of a delay in screening programs, fear of detection, and fear of interruption in new screening programs are among the most important fears at the diagnostic level. At the treatment level, patients are afraid of weakening immune system due to chemotherapy, delay in immediate treatment, and interruption in treatment schedules. Fear of infection, weak immunity against the virus, travel and caution among caregivers, as well as fear of supporting family and others, fear of social isolation and fear of infection are among the most important fears of the individual in this period (Moraliyage et al., 2021). Guven found more than 90% of cancer patients had moderate to severe fear of COVID-19 (Guven et al., 2020). Finally, some studies did not confirm the increase in psychological distress in cancer patients during the COVID-19 pandemic (Musche et al., 2020; Büssing et al., 2021; Hill et al., 2021; van de Poll-Franse et al., 2021; van Gorp et al., 2021).

### Effects of Psychological Disorders on the Patient

What is clear is that dealing with these distresses is a high priority, because it can affect the health of individual and society. Vanni et al., who studied the effect of COVID-19 pandemic on suspected breast cancer patients, reported that COVID-19 related anxiety affects the patient's decision-making processes regarding the treatment (Vanni et al., 2020). To deal with this crisis, only 1.6% of patients sought psychiatric help to find a solution (Wang et al., 2020a).

The results of a retrospective study of 160 patients with or suspected of breast cancer showed that COVID-19 related anxiety could discourage treatment (Vanni et al., 2020). A survey of 1,079 patients with multiple myeloma showed that they have concern about the future and events ahead, worries about family, friends and relatives, and also have paternal irritation, feelings of sadness, anger, fear, loneliness, and problems communicating with their spouses during the COVID-19 pandemic (Sweeney and Ahlstrom, 2020).

## Discussion

COVID-19 pandemic is currently the most important health problem worldwide. In this review study, the prevalence, causes and outcomes of psychological disorders among cancer patients during the COVID-19 pandemic were reported. The results of this study showed that the rate of these disorders has an upward trend during the pandemic. A review of similar studies in the pre-pandemic period shows that one in five patients experienced high level of psychological distress (Dehghan et al., 2021). Although in line with the Norton study, which showed that younger age and advanced disease are associated with higher level of distress (Frey et al., 2020), this study elucidated the factors that lead to an increase in patient distress following the COVID-19 pandemic. The COVID-19 related anxiety is a logical and expected reaction to the current situation. Although this fear and anxiety affects many people, it can confuse cancer patients to the point that they refuse to continue treatment for the fear of infection and worsening of their condition (Ornell et al., 2020). Also, since the end of this pandemic is unknown, this action can endanger the health of this group of patients, so it seems that using psychological interventions in the current situation is one of the main priorities for cancer patients, even though in the pandemic situation, patients have less access to psychiatrists and only have limited contact with oncologists.

In addition to anxiety, during a pandemic, due to the recommendation to observe social distancing and quarantine, people communicate less with each other and the risk of depression in the general population is increased (Benke et al., 2020). Besides cancer diagnosis which is a stressful process, accompanied by different psychological distresses, the choice of right treatment protocol, proper approaches for increasing individual's knowledge, reduction of side effects and finally the exposure of the individual and his family with this dilemma are all considered as critical choices which have been exacerbated by Covid-19 pandemic. Therefore, it is recommended that psychologists and social workers monitor patients in a situation where the pandemic is under control, and support them according to the existing conditions so that, their depression would be under control (Chen et al., 2021a). This is an important intervention, because not recognizing depression and anxiety can endanger patients' survival (Wang et al., 2020b). In general, in order to provide the best diagnostic and therapeutic services, each country should develop clear protocols based on the pandemic conditions in the country, in order to avoid unnecessary surgeries. Also, using multi-specialized teams and providing sufficient counseling, the treatment of different patients should not be interrupted, because even under normal circumstances, fear and anxiety can change the course of disease and interfere with the treatment process (Chen et al., 2021a).

Different approaches have been recommended to reduce the variety of distresses experienced by cancer patients, the most important of which is accurate and timely information, empowering decision making, supporting coping approaches, empowering decision making, prioritization of treating patients considering the type of malignancy and the choice of treatment protocol, assessment of treatment advantages besides the probability of being affected by Covid-19, the change of standard treatment protocol to reduce the exposure, management of side effects related to the treatment and the reduced need for face to face attendance, guaranteeing the medical services and effective communication with patient and his family (Ballal et al., 2020; Bersanelli, 2020; Hanna et al., 2020). One of the most significant approached to eliminate some of these challenges is to apply telemedicine technologies as a safe and yet effective approach applied throughout the world. Telemedicine can increase people's adaptation to treatment by reducing fear and anxiety (Al-Quteimat and Amer, 2020). Therefore, the use of this approach and also behavioral psychotherapy through video conferencing, which have already proven their effectiveness in improving the quality of life of cancer patients, is recommended (Akula et al., 2020). Canada provides psychological support packages using text messages and cost-effective, population-based interventions to reduce the severity of distress in cancer patients during the COVID-19 pandemic (Agyapong et al., 2020).

Despite its low accessibility in some regions throughout the world, especially low-income and countries with limited resources, in countries that this technology is available, some patients avoid it (Chávarri-Guerra et al., 2021). On the other hand, it is difficult to discern which patients benefit the most from either face to face or telemedicine services. Finally, some scholars have argued that telemedicine is regarded as the main reason behind patients' distress and reduction of the quality of psychological health which contributes to higher problems in some cases.

Although background variables such as age and gender are involved in emergence of psychological distresses during Covid-19 pandemic period, but most of the distresses have occurred due to dysfunctions in oncologic services, changes in routine programs, and delays in treatment protocols (Chen et al., 2020, 2021a; Gultekin et al., 2021; Nardone et al., 2021). Although the modification of treatment protocols in Covid-19 pandemic have been observed in most countries throughout the world, but extensive variation is observed within treatment plans in different regions which is variable from 25- up to 90% according to the literature (Al-Quteimat and Amer, 2020; Altin et al., 2020; Kuderer et al., 2020; Martinelli and Garbi, 2020).

According to the reports made by the scholars, health care system plays a significant role in patients' psychological well-being. Some nations have devised and modified guidelines to reduce the mental distress and to yield the best positive results and they updated this health care system based on the facilities available in the country and the status of the pandemic. These guidelines have been designed to facilitate patient's conditions, the progression stage of his illness and the administration of the illness during covid-19 pandemic period and reduces their confusion (Akula et al., 2020; Raghavan et al., 2020). Hus, the governments are not only responsible for ensuring the accessibility and provision of proper services, but play a significant role in patients' psychological well-being.

This study has some limitations that need to be addressed. We tried to include all relevant studies in this subject. However, as COVID-19 data reporting is a growing process and we analyzed studies until June 2021. The use of different tools to measure depression in different studies reduces the possibility of comparison, and also may not indicate the true prevalence of depression in the target community. In addition, the small number of studies conducted on this issue reduces the generalizability of findings. On the other hand, due to the limited resources in this field, quality assessment was not performed and all studies that met the inclusion criteria were included in the study.

## Data Availability Statement

The original contributions presented in the study are included in the article/Supplementary Material, further inquiries can be directed to the corresponding author/s.

## Author Contributions

All authors listed have made a substantial, direct and intellectual contribution to the work, and approved it for publication.

## Conflict of Interest

The authors declare that the research was conducted in the absence of any commercial or financial relationships that could be construed as a potential conflict of interest.

## Publisher's Note

All claims expressed in this article are solely those of the authors and do not necessarily represent those of their affiliated organizations, or those of the publisher, the editors and the reviewers. Any product that may be evaluated in this article, or claim that may be made by its manufacturer, is not guaranteed or endorsed by the publisher.

## Appendix 1

Search strategy in included database.

WOS, KJD, RSCI, SCIELO Timespan=All years

# 4 #3 AND #2 AND #1

# 3 TOPIC: (COVID-19) OR TOPIC: (covid)

# 2 TOPIC: (Neoplasms) OR TOPIC: (cancer)

# 1 TOPIC: (Depression) OR TOPIC: (Mental Disorders) OR TOPIC: (Psychiatric Illness) OR TOPIC: (Anxiety)

PubMed

(((((((“Depression”[Mesh]) OR ((Depressions[Title/Abstract]) OR (Emotional Depression[Title/Abstract]))) OR ((Depressions[Title/Abstract]) OR (Emotional Depression[Title/Abstract]))) OR ((“Mental Disorders”[Mesh]) OR ((((Mental Illness[Title/Abstract]) OR (Mental Disorder[Title/Abstract])) OR (Psychiatric Illness[Title/Abstract])) OR (Psychiatric Disorder[Title/Abstract])))) OR ((Psychological Distress[Title/Abstract]) OR (“Psychological Distress”[Mesh]))) OR ((Anxiety[Title/Abstract]) OR (“Anxiety”[Mesh]))) AND ((“Neoplasms”[Mesh]) OR (((((Neoplasia[Title/Abstract]) OR (Neoplasm[Title/Abstract])) OR (Tumor[Title/Abstract])) OR (Cancer[Title/Abstract])) OR (Malignancy[Title/Abstract])))) AND (((“COVID-19”[Mesh]) OR (((((((COVID-19[Title/Abstract]) OR (COVID 19 Pandemic[Title/Abstract])) OR (Coronavirus Disease 2019[Title/Abstract])) OR (Infection, SARS-CoV-2[Title/Abstract])) OR (COVID-19 Virus Infections[Title/Abstract])) OR (SARS Coronavirus 2 Infection[Title/Abstract])) OR (COVID 19[Title/Abstract]))))

Scopus

(TITLE-ABS-KEY(covid) AND TITLE-ABS-KEY(cancer) AND TITLE-ABS-KEY((Depression) OR (Mental Disorders) OR (Psychiatric Illness) OR (Anxiety)))
