# Supplementary material for: Psychological Distress Among Cancer Patients During COVID-19 Pandemic in the World: A Systematic Review
Source: Front Psychol. 2021 Sep 28;12:682154. doi: 10.3389/fpsyg.2021.682154 (PMC8506116; doi:10.3389/fpsyg.2021.682154)
Supplement: Supplementary file 1 [file Table_1.doc]

**Table 1: Characteristic of the included studies**

| **Author/ Year** | **Location** | **Design** | **Sample size** | **Cancer**  **Type** | **Participant age** | **Questionnaire** | **Main results** |
| --- | --- | --- | --- | --- | --- | --- | --- |
| Agua/ 2021  (8) | Spain | Cross-sectional | 2779 | N/M | 40.2% were over 50 years old | Kessler (K-6) scale | 33.5% of patients experienced clinical stress levels. Stress levels were significantly higher in young patients and women. |
| Bargon/ 2020  (9) | Netherlands | Cohort | 1595 | Breast | N/M | quality of life (EORTC) questionnaires | A significant decrease in emotional functioning was seen in cancer patients, and 48% of the patients reported moderate to severe loneliness. |
| Büntzel/ 2021(10) | Germany | Cross-sectional | 433 | Head and neck  Breast  Other | Between 50 -60 | Researcher made instrument | 42.9% of the cancer patients experienced strong or very strong mental stress during pandemic. |
| Büssing/ 2020  (11) | Germany | Cross-sectional | 292 | Larynx  Breast  Prostate | Mean± SD:  66.7±10.8 | WHO-Five Well-being Index (WHO5)  Meaning in Life Questionnaire (MLQ)  Spiritual and Religious Attitudes to cope with illness (SpREUK-15)  Gratitude/Awe scale (GrAw-7)) | Patients´ wellbeing scores were in the lower range which show depressive status of the sample. Most of the participants reported concerns regarding the danger and the course of the COVID-19 and complicated course of disease. |
| Büssing/ 2021  (12) | Germany | Cross-sectional | 295  221  (two time periods) | Prostate  Larynx  Nasal/paranasal  Breast  Rectal | Sample 1: 66.7 ± 10.8  Sample 2: 66.0 ± 10.2 | Perceptions of Change Scale  WHO-Five Well-being Index (WHO-5)  numeric analogue scales (NAS)  Meaning in Life Questionnaire (MLQ) | The proportion of patients whose daily lives were affected by cancer or COVID-19 was similar.  Patients’ wellbeing and meaning in life scores were similar in two waves.  Comparing both samples from wave 1 and wave 2, fears and worries were not significantly different among cancer patients. |
| Chaix/2020  (13) | France | Cross-sectional | 1771  Asthma: 497  Breast cancer: 360  Depressive disorder: 459  Migraine: 455 | Breast cancer  Other at risk groups | Mean± SD:  32.4±13.39  (Total participants) | Psychological Distress Inventory (PDI) | Prevalence of psychological distress (PDI≥ 14) was 34% for breast cancer patients. Sex, unemployment and depression were significantly associated with higher score of PDI. PDI score was higher in the regions with higher prevalence of COVID-19. |
| Chapman/ 2020  (14) | UK | Cross-sectional | 234 | Breast | Mean: 51  Range: 27-78 | Functional Assessment of Cancer Therapy-Cognitive Scale  Rumination Response Scale  Hospital Anxiety and Depression Scale  Penn State Worry Questionnaire  Modified Self-Report-Generated Charlson Comorbidity  Work Limitations Questionnaire | Job insecurity was significantly associated with depression, anxiety and lower cognitive function. |
| Chen/ 2020  (15) | China | Cross- sectional | 326 | Breast cancer  Digestive system cancer Lung cancer Others | 18-60: 61.7%  ≥61: 38.3% | Fear of Progression Questionnaire-Short Form (FoP-Q-SF)  Self-Rating Anxiety Scale (SAS)  Self-Rating Depression Scale (SDS) | 86.5 % of the patients stated fear of disease progression, 67.5% reported anxiety and 74.5% showed depression.  Multiple linear regression analysis showed that treatment delay, treatment interruption, deep concern about Covid-19 and lung cancer diagnosis were significantly associated with FOP-Q-SF scores.  Treatment delay, treatment interruption, deep concern about COVID-19 and lung cancer diagnosis were positively and educational level was negatively correlated with SAS scores.  Treatment delay, treatment interruption, deep concern about COVID-19 and unmarried status were positively and higher income was negatively correlated with SDS scores. |
| Chen/2021  (16) | China | Cross-sectional | 834 | Breast | <46: 34.9%  ≥46: 65.1% | Patient Health Questionnaire (PHQ-9)  Generalized Anxiety Disorder (GAD-7)  Insomnia Severity Index (ISI) questionnaires | The prevalence of depression, anxiety, and insomnia was 21.6%, 15.5%, and 14.7%.  According to multivariate analysis, living alone, comorbidity and deterioration of breast cancer were associated with more severe symptoms of depression, anxiety and insomnia.  Changed treatment plan was associated with depression and anxiety. |
| Chen/ 2021  (17) | US | Cross-sectional | 100 | Gynecological cancer | Mean: 60  Range: 19-86 | Selected questions from validated surveys | A delay in cancer care was associated with a 4-fold increased rate of anxiety. Race was not significantly associated with increased financial distress, cancer worry, or anxiety. |
| Chia/ 2021  (18) | Singapore | Qualitative | 16 patients  14 caregivers | Colon  Lung  Lymphoma  Prostate Pancreas Stomach Adrenal Brain Nose  Germ cell tumor | Mean± SD:  60.1±14.4 | Interview | Vulnerability and fear were one of the psychological responses of cancer patients in the COVID-19 pandemic. Cancer, immunosuppressed status and risk of exposure when visiting the hospital increased patient’s fear. |
| Cui/ 2020  (19) | China | Cross-sectional | 207 patients  684 nurses | Breast | <40: 20.8%  40-55: 51.2%  >55: 28.0 | Patient Health Questionnaire (PHQ-9)  Generalized Anxiety Disorder scale (GAD-7)  Insomnia Severity Index (ISI)  Impact of Event Scale-Revised (IES-R) | More than half of breast cancer patients reported abnormal status based on PHQ-9, GD-7 and ISI that was comparable to frontline female nurses.  Occurrence of insomnia and anxiety among breast cancer patients were more than for frontline female nurses. |
| Dehghan/ 2021  (20) | Iran | Cross-sectional | 184 | Breast  Gastrointestinal  Lymphoma  Bone marrow  Genitalia | Mean± SD:  52.85±14.66 | Corona Disease Anxiety Scale (CDAS)  The Freiburg Mindfulness inventory-Short Form (FMI-SF)  Spiritual health scale | 61.4 % of participants reported moderate to severe anxiety. No significant relationship was observed between COVID-19 related anxiety and spiritual health. |
| Frey/ 2020  (21) | US | Cross-sectional | 603 | Ovary | Mean: 58 | Cancer Worry Scale Hospital Anxiety and Depression Scale | More than half of patients reported borderline or abnormal anxiety level and 26.5% reported borderline or abnormal depression.  Younger age, immunocompromised status, and delay in cancer care were associated with significantly higher level of cancer worry, anxiety, and depression. |
| Ghallagher/2020  (22) | UK | Cross-sectional | 518 | Breast  Prostate  Blood  Other | Breast:  Mean± SD:  63.7±11.49  Prostate: 68.4±8.21  Blood: 59.2±14.54  Other: 64.6±11.87 | General Health Questionnaire  Loneliness: Single item | The risk of depression in breast, prostate and blood cancer increased.  Feeling of loneliness increased the risk of depression up to 4.5 fold. |
| Gultekin/ 2020  (23) | 16 European countries | Cross-sectional | 1388 | Gynecology | Mean: 55  Range: 18-89 | Hospital Anxiety & Depression Scale questionnaire (HADS)  COVID-19-related questionnaire | 35.3% of patients reported abnormal anxiety level and 30.6% reported abnormal depression status.  Gynecological cancer patients experienced significant worry about progression of their disease due to modifications of care, delay or cancellation of oncology treatment or follow-up in the COVID-19 pandemic.  Older age was the only risk factor for greater fear of COVID-19 compared to cancer |
| Guven/ 2020  (24) | Turkey | Cross-sectional | 195 | Brest  Colorectal  Lung  Genitourinary  Head and neck  Other | Mean:59  Range: 20-82 | Researcher made instrument | More than 90% of patients had moderate to severe fear of COVID-19. |
| Hamlish/ 2021  (25) | USA | Cross-sectional | 633 | Breast | Mean± SD:  47.92±10.86 | Researcher- made instrument | White participants experience more stress than blacks, and often worry about interruptions or delays in care, treatment, worsening of the disease, COVID-19, and general health. |
| Han/ 2021  (26) | China | Cross-sectional | 111 | Prostate  Breast  Lung and bronchus  Colon and rectum | Mean± SD:  56.6±9.62 | symptom checklist 90 (SCL‐90) | Somatization, interpersonal sensitivity, depression, anxiety, hostility, phobic anxiety, paranoid ideation were significantly higher than pre COVID-19 pandemic. |
| Hill/ 2021  (27) | USA | Cross-sectional | 100 | Ovary | Mean± SD:  55.03±12.03 | Intolerance of Uncertainty Scale  Fear of COVID-19 Scale (FCS)  Depression Anxiety Stress Scales (DASS-21) | Women with ovarian cancer did not experience more distress during the COVID-19 pandemic. |
| Hilbert-Williams/ 2021  (28) | UK | Cross-sectional | 144 | Brain/CNS Breast  Gynecological Hematological Head & Neck  Lower GI / Bowel Lung  Pancreatic Prostate Sarcoma Upper GI  Urology / Bladder | Mean± SD:  57.63±11.88 | Supportive Care Needs Survey  Depression, Anxiety and Stress Scales | After the onset of the pandemic, patients' anxiety was significantly lower, stress was significantly reduced, and depression was unchanged. |
| Irusen/ 2021  (29) | South Africa | Cross-sectional | 60 | Prostate | Mean± SD:  63.2±5.9 | State-Trait Anxiety Inventory (STAI-S)  the Connor-Davidson Resilience (CD-RISC) scale  Scale of Perceived Social Support (MSPSS) | COVID-19 related anxiety was very low and was not clinically significant. There was no correlation between state anxiety (STAI-S) and COVID-19 anxiety (CAS). |
| Kizilkan/ 2021  (30) | Turkey | Cross-sectional | 24 | Prostate | Mean± SD:  64.8±7.5 | State- Trait Anxiety Inventory [STAI]  Beck Depression Inventory [BDI] | A significant inverse relationship was observed between age and STAI score.  Patients with prostate cancer during the COVID-19 pandemic reported mild depression. |
| Koinig/ 2021  (31) | Australia | Cross-sectional | 240 | Breast cancer  Chronic lymphocytic leukemia (CLL)  Multiple myeloma (MM)  Myelodysplastic syndromes (MDS)  Prostate cancer Lung cancer  Squamous cell carcinoma of head and neck (HNSCC)  Acute myeloid leukemia (AML)  Non- Hodgkin's lymphoma (NHL) | Mean± SD:  67±13 | 13- item survey on COVID- 19’s impact on cancer patient's distress and everyday life  European Organization  for Research and Treatment of Cancer Quality of Life Questionnaire (EORTC QLQ- C30) | Young patients, women and people with mental disorders experience more distress during the COVID-19 pandemic.  Psychological distress occurred in 14% of patients due to COVID-19 pandemic. |
| Košir / 2020  (32) | UK | Cross-sectional | 177 | N/M | Mean± SD:  29.33±6.17 | Patient Health Questionnaire for Depression and Anxiety (PHQ-4) | More than a third of participants reported an increase in psychological distress. Sixty-two percent of participants said their anxiety was higher than before the pandemic. |
| Letaief-Ksontini/ 2020  (33) | Tunisia | Cross-sectional | 91 | N/M | 64.8% were over 40 years old | Hospital Anxiety and Depression Scale (HADS) | The incidence of depression in cancer patients was 29.7% in COVID-19 pandemic. 66.7% of patients had mild depression, 25.9% moderate and 7.4% had severe depression.  The incidence of anxiety was 69.2%.  Multivariate analysis of logistic regression showed that family support during the COVID-19 pandemic was negatively and pain was positively correlated with the level of anxiety. |
| Lou/ 2020  (34) | China | Randomized control trial (RCT) | 58 | Head and neck | Mean± SD:  46±8.3 | Self-Report Symptom Inventory  Symptom Check-List90 (SCL-90)  Self-Rating Depression Scale (SDS)  Self-Rating Anxiety Scale (SAS) | Compared to national norm, depression, anxiety, hostility and phobic anxiety increased in COVID-19 pandemic. |
| Levy/ 2021  (35) | Israel | Mixed methods | 408 | Hematology | Mean± SD:  60±14 | Patient Health Questionnaire 9 (PHQ-9) | Mild, moderate, moderately sever and severe depression were in 35.2%, 24.5% 12.1% and 5.7% of the participants respectively. |
| Mahl/ 2020  (36) | Brazil | Cross-sectional | 31 | Head and neck | Mean: 55 | self-perception of anxiety and sadness, fear of COVID-19 infection, cancer-related complications during social isolation, self-medication, diagnosis of COVID-19, and death and Self-reported anxiety and sadness | Fear of COVID-19 infection and feelings of anxiety and sadness were 41.9%, 71.0%, and 45.2% respectively. |
| Massicotte/ 2021  (37) | Canada | Cross-sectional | 36 | Breast | Mean±SD: 53.6±10.9 | COVID-19 Stressors Questionnaire  Insomnia Severity Index (ISI)  Hospital Anxiety and Depression Scale (HADS)  Severity Subscale of the Fear of Cancer Recurrence Inventory (FCRI) | Cancer patients experience a significant number of stressors in the pandemic, which are associated with greater level of anxiety, depression and insomnia and fear of cancer recurrence. |
| Miaskowski/2020  (38) | US | Cross-sectional | 187 | Breast  Gastrointestinal  Lung  Malignant melanoma  Gynecological  Prostate  Multiple cancer | Mean±SD: 63.3±10.9 | 22-item Impact of Event Scale-Revised (IES-R)  Social Isolation Scale (SIS)  Spielberger State-Trait Anxiety Inventories (STAI-S, STAIT)  General Sleep Disturbance Scale (GSDS)  Lee Fatigue Scale (LFS, which assessed levels of morning and evening fatigue and morning and evening energy)  Attentional Function Index  Brief Pain Inventory | The incidence of depression, anxiety, sleep disturbance, evening fatigue, cognitive impairment and pain were 71.2%, 78%, 78% 55.9%, 91.5% and 75.9% respectively.  Patients in the stress group, reported higher levels of comorbidity. Fewer years had passed since cancer diagnosis. They were more likely to make a diagnosis of depression and report a lower functional status score. |
| Miaskowki/ 2021  (39) | US | Cross-sectional | 606 |  |  |  | 66% of patients reported loneliness.  Multivariate analysis of logistic regression showed that being unmarried, having higher levels of social isolation, and higher levels of anxiety and depressive symptoms were associated with membership in the lonely group. |
| Musche/ 2020  (40) | Germany | Case-control | 150 patients  150 controls | Bone cancer, cartilage tumor, soft-tissue sarcoma  Breast  Cancer of the central nervous system  Cancer of the gastrointestinal tract  Cancer of the eye  Head and neck cancer  Leukemia or Lymphoma  Lung cancer  Skin cancer  Thyroid cancer  Urogenital cancer | <45->75 | European Quality of Life 5 Dimensions 3 Level Version (EQ-5D-3L)  Generalized-Anxiety-Disorder (GAD-7) | Cancer patients and the control group reported an increase in COVID- 19 related fear and the difference between the two groups was not statistically significant. |
| Nardone/2021  (41) | Italy | Cross-sectional | 458 | Gastrointestinal    Brain  Breast  Lung  Prostate  Head and Neck | Mean: 63.9 | State trait anxiety inventory scale  Symptom Distress thermometer   Beck Depression Inventory v.2 | The prevalence of psychological disorders in patients undergoing radiation therapy is high. |
| Ng/2020  (42) | Singapore | Cross-sectional | 624 | Breast  GI and hepatobiliary tract Lung Ovarian/endometrial/cervix Lymphoma/hematologic Renal/bladder/prostate/urologic Head and neck Brain Sarcoma Multiple cancers | Mean± SD:  57.2±12.2 | Generalized Anxiety Disorder-7  Self-reported fears related to COVID-19 | The prevalence of anxiety was 19.1%  Patients who were no graduates and married, were more anxious. |
| Ng/2020  (43) | Hong Kong | Cross-sectional | 117 | Breast  Colorectal | Cancer patients:  Mean± SD:  52.96± 8.34  Control: 57.78±8.77 | Hospital Anxiety and Depression Scale (HADS)  Chinese Brief COPE  Chinese Short Health Anxiety Inventory (SHAI) | Cancer patients reported higher COVID-19-related catastrophizing, increased general health anxiety, greater use of avoidance coping, less use of problem-focused and emotion-focused coping, and lower HADS-anxiety during the pandemic compared with healthy controls. |
| Papautsky/ 2021  (44) | USA | Cross-sectional | 633 | Breast | Mean± SD:  47.93±10.95 | Researcher made | There was a significant negative correlation with trust and worry.  Active treatment, immunocompromised status, and delays in care were related to greater level of worry in cancer patients. |
| Pigozzi/ 2021  (45) | Italy | Cross-sectional | 474 | Breast  Lung  Colorectal  Hematologic  Pancreas  Gynecologic  Prostate  Head and neck  Kidney  Stomach | Mean:62  Range: 20-97 | Emotional Vulnerability Index (EVI) | The EVI increased during the pandemic up to 44%. There were a significant association between female gender and type of therapy with the vulnerability. |
| Rodrigues-Oliveira/ 2021  (46) | Brazil | Cross-sectional | 50 | Head and neck | Mean± SD:  58.8±9.89 | Hospital Anxiety and Depression Scale (HADS)  Distress thermometer (DT) | The prevalence of both anxiety and depression were 22%. |
| Romito/ 2020  (47) | Italy | Cross-sectional | 77 | Lymphoma | Mean: Range: 56.6 | Impact of Event Scale-Revised (IES-R) Hospital Anxiety and Depression Scale (HADS) | The incidence of psychological disorders were as follow:  Anxiety (36%), depression (31%), post-traumatic stress disorder (PTSD) (36%).  Women and younger patients were found to be more vulnerable to anxiety and PTSD. |
| Salehi/ 2021  (48) | USA | Mixed methods | 104 patients  50 providers | Lymphoma/leukemia or blood malignancies  Colorectal  Pancreatic  Lung  Prostate | Mean: 66.8  Range: 27-97 | Researcher made  interview | Only concerns regarding vulnerability to infection were statistically significant before and after pandemic. |
| Seven/ 2021  (49) | Turkey | Qualitative | 18 | Breast | Mean± SD:  51±5.9 | Interview | Anxiety, feeling oversensitive, emotional burnout, having psychosomatic symptoms, contestant check symptoms and worry about children’s health are among the most important experience of breast cancer during COVID-19 pandemic. |
| Schellekens/ 2020  (50) | Netherlands | Cross-sectional | 233 | N/M | <30-≥70 | The 12-item survey including questions regarding loneliness, fear of infection and dying from COVID-19 | 50.5% of patients were afraid of COVID-19 and 58% were afraid of being admitted to the intensive care unit. 36.3% of patients felt lonelier than before the pandemic. |
| Sigorski/ 2020  (51) | Poland | Cross-sectional | 306 | Gastrointestinal cancer  Lung cancer  Melanoma and sarcoma  Breast cancer  Urogenital cancers | Mean: 63  Range: 25-67 | Numerical Anxiety Scale  The State-Trait Anxiety Inventory  The Mini-Mental Adjustment to Cancer | COVID-19 related fear and anxiety are significantly lower than cancer related anxiety. The highest anxiety level was in breast cancer patients and the lowest was reported in lung cancer patients. |
| Sun/ 2021  (52) | China | RCT | 62 | Lung | Intervention:  Mean± SD:  61.6±8.16  Control: 60.19±7.10 | Self‑Rating Anxiety Scale (SAS)  Self‑Rating Depression Scale (SDS) | Rate of anxiety in patients with lung cancer reached 35.71%, while the rate of depression reached 51.43%. |
| Swainston/2020  (53) | UK | Cross-sectional | 234 | Breast | Mean: 51 | Rumination Response Scale  Hospital Anxiety and Depression Scale  Penn State Worry Questionnaire  Modified Self-Report-Generated Charlson Comorbidity | Change in oncology treatment had a significant effects in COVID-19 related emotional vulnerability, general anxiety and depression.  Rumination and worry were associated with depression.  Greater COVID-19 emotional vulnerability related to higher level of depression. |
| Sweeney/ 2020  (54) | US | Cross-sectional | 1079 | Multiple myeloma | N/M | distress screening tool | Psychosocial concerns were as follow:  worrying about the future (46%), worry about family, children, and/or friends (44%), feeling irritable (37%), feeling sad or depressed (30%), feeling nervous or afraid (25%), feeling lonely or isolated (20%), and relationship problems with your spouse/partner (11%). |
| Van de Poll-France  (55) | Netherlands | Cross-sectional | Patients: 4094  Matched normal participants: 977 | N/M | Male: 63.0±11.1  Female: N/M | Hospital Anxiety and Depression Scale  De Jong Gierveld short scales | Anxiety and depression were not statistically significant between the two groups, but the normal group expressed more loneliness. |
| Van Gorp/ 2020  (56) | Netherlands | Cross-sectional | 799 | Hematological cancer  Other | Mean± SD:  9.4±4.9 | distress thermometer for parents (DT-P) | The psychological distress of children with cancer during the COVID-19 pandemic is no different from the past. |
| Vanni/ 2020  (57) | Italy | Retrospective | 160 | Breast | Mean: 59.5  Range: 39-77 | Interview | COVID-19-related anxiety could affect decision making process in patents. |
| Wang/2020  (58) | China | Cross-sectional | 6213 | Nasopharyngeal  Digestive  Lung and mediastinum  Nervous system  Soft tissue sarcoma  Bone  Breast  Skin  Lympho hematopoetic  Gynecological  Head and neck  Urinary system | Mean±SD: 50.57±13.28 | Generalized Anxiety Disorder-7 (GAD-7)  Patient Health Questionnaire-9 (PHQ-9)  Brief Symptom Inventory (BSI)  Impact of Events Scale-Revised (IES-R) | The incidence of psychological disorders were as follow: depression (23.4%), anxiety (17.7%), PTSD (9.3%), and hostility (13.5%). |
| Wong/ 2021  (59) | Malaysia | Cross-sectional | 631 | Breast  Prostate  Bladder  Ovarian  Cervical  Nasopharyngeal  Lymphoma  Colon  Appendix | Mean± SD:  56.98±11.21 | Hospital Anxiety and Depression Scale (HADS) | The proportion of participants with anxiety and depression was 29.0 and 20.9% respectively. |
| Yang/ 2021  (60) | China | Cross-sectional | 609 | N/M | 81.36% were 18-60 years old | Self-rating Anxiety Scale (SAS) | 15.15% of participants reported anxiety.  Age >60 years old, the farmer occupation, and higher sleep disorder were risk factors for anxiety.  70.73 % of participants reported fear of tumor aggression. |
| Yildirim/ 2021  (61) | Turkey | Cross-sectional | 595 | Breast  Ovary  Colon  Gastric | Mean± SD:  50.48±14.89 | Beck Depression Inventory (BDI) Beck Anxiety Inventory (BAI) | Both BDI and BAI scores increased in the cancer patients.  The increase was positively correlated with the disruption of cancer treatment.  Depression and anxiety were more common in female patients. |
| Zhang/ 2021  (62) | China | Cross-sectional | 141 | Lung Breast  Colorectal Gastric | ≤60:85 (60.3%)  >60: 56 (39.7%) | hospital anxiety and depression scale (HADS) | Prevalence of anxiety and depression increased in cancer patients with advanced refractory disease.  The prevalence of anxiety was high in cancer patients aged 60 years or older.  Anxiety was associated with advanced incurable cancer.  There were significantly increased scores of anxiety in patients with lung cancer. |

**🞾N/M: Not Mentioned**
